# Surface Modification of Bamboo Fibers to Enhance the Interfacial Adhesion of Epoxy Resin-Based Composites Prepared by Resin Transfer Molding

**DOI:** 10.3390/polym11122107

**Published:** 2019-12-15

**Authors:** Dong Wang, Tian Bai, Wanli Cheng, Can Xu, Ge Wang, Haitao Cheng, Guangping Han

**Affiliations:** 1 Key Laboratory of Bio-based Material Science and Technology (Ministry of Education), Northeast Forestry University, Harbin 150040, China; sdzcwangdong@nefu.edu.cn (D.W.); baitian@nefu.edu.cn (T.B.);; 2 International Centre for Bamboo and Rattan, Beijing 100102, China

**Keywords:** bamboo fiber, interfacial bonding, polymer–matrix composites, resin transfer molding

## Abstract

Bamboo fibers (BFs)-reinforced epoxy resin (EP) composites are prepared by resin transfer molding (RTM). The influence of BFs surface modification (NaOH solution or coupling agents, i.e., KH550 and KH560) on interfacial properties of BFs/EP composites is systematically investigated. The synergistic effect of hydrolysis, peeling reaction of BFs, and the condensation reaction of hydrolyzed coupling agents are confirmed by FTIR. Scanning electron microscopy (SEM) and atomic force microscopy (AFM) reveal that the interfacial compatibility of NaOH- and silane-modified BFs/EP composites was significantly improved. KH550-modified BFs/EP composite renders optimal tensile, flexural, and impact strength values of 68 MPa, 86 MPa, and 226 J/m. The impact resistance mechanism at the interface of BFs/EP composites was proposed. Moreover, the dynamic mechanical properties, creep behavior, and differential scanning calorimetry of BFs/EP composites have also been carried out to understand thermal stabilities. Overall, the surface-modified BFs-reinforced EP composites exhibited superior interfacial bonding.

## 1. Introduction

Epoxy resin (EP) is an important class of polymer oligomers with strong cohesion and dense molecular structure. The curing system of EP contains multiple reactive groups, such as epoxy groups, hydroxyl groups, ether bonds, and amine bonds, resulting in high reactivity and superior bonding strength. Furthermore, EP is widely used as a matrix resin for different high-end structural materials by using a variety of fabrication techniques, such as casting, and injection molding [1,2,3,4,5]. However, cured EP renders high internal stress, poor fatigue resistance, high brittleness, poor toughness, inferior impact resistance, and poor weather resistance. In addition, the high surface energy limits the utilization of EP in high-performance structural components [1,4]. Therefore, it is of utmost significance to carry out structural modifications to improve the toughness and reduce the brittleness of EP for a wide range of applications [6,7].

Various methods based on the physicochemical treatment of matrix and filler materials are used to obtain high-performance composites [8,9,10]. In recent years, natural fiber (NF)-reinforced EP composites have garnered significant research attention for structural applications [11,12,13,14,15,16]. These materials can be applied to the automotive industry, sports goods, and aerospace where light-weight components are needed [5]. NF can significantly improve the mechanical properties of the EP matrix and different NFs, such as bamboo, flax, and cotton, exhibit low cost, low density, and high specific strength [17,18,19]. The incorporation of NF can transfer or distribute a load of EP matrix to the fibers and, thereby, effectively solve the problem of stress concentration within the material [20]. Therefore, a large number of studies have focused on NF reinforced composites [13,21]. For instance, Zafeiropoulos et al. detail the surface modification of fibers by designing the interfacial between the NF and the matrix in the manufacture of NF composites to achieve the maximum performance of the NF composites [22]. Pickering et al. provide a useful overview of natural fibers whereby fibers affect the mechanical performance of the matrix [16]. Yan et al. have modified the coir fiber (CF), with 5 wt.% NaOH, to prepare a CF/EP composite. The results reveal that the alkali-treated CF renders a cleaner and rougher surface than the as-prepared CF, improving the interfacial properties of the fiber and EP. Consequently, the compressive and flexural strength of CF/EP composite is significantly enhanced and the damping ratio is effectively reduced [17].

As a prominent member of the NF family, bamboo has gained increasing research attention due to its high toughness and simple processing [23,24], making it a desirable candidate for fiber-reinforced resin composites [25,26]. However, similar to other NFs, bamboo fibers (BFs) are also rich in hydroxyl and polar groups, resulting in high moisture absorption, which negatively influences the interfacial bonding with hydrophobic matrix [27,28]. Thus, it is of utmost importance to improve the interfacial properties between natural filler and matrix to enhance the physical and mechanical properties of the composite.

In general, the interfacial properties can be tuned by carrying out suitable surface modifications via chemical processes [22,29,30,31]. For instance, a low-cost and easy-to-operate sodium hydroxide (NaOH) treatment is carried out to effectively dissolve the lignin and hemicellulose, which improves the interfacial adhesion between BFs and resin [32,33]. It has been reported that the alkali-treated fibers render superior interfacial properties to the matrix [34]. For example, Baillie and Zafeiropoulos et al. modify the surface properties of the fiber by pretreating with NaOH solution to obtain the NF reinforced composites with good interfacial adhesion [19]. However, different material systems require optimal NaOH process parameters to achieve maximum performance. Moreover, the coupling agent, as an amphiphilic substance, significantly improves the interfacial adhesion by enhancing the compatibility of two substances [18]. The 3-aminopropyltriethoxysilane (KH550) and γ-(2,3-epoxypropoxy)propytrimethoxysilane (KH560) are used to improve the bond strength between the NF and the matrix. Wu et al. have employed KH550 to modify interfacial adhesion of carbon fiber/methylphenylsilicone resin composite and demonstrated an increase of 31.12% in the impact strength [35]. It is worth noting that conventional coupling agents, such as 1,3-diaminopropane and hexamethylenediamine, contain only two reactive groups per molecule [15,36], resulting in limited or negligible improvement in interface properties after modification. Even though the influence of BF addition on structural properties of resin has been studied, the detailed exploration of BF reinforced epoxy composites, prepared by resin transfer molding (RTM), has not been carried out yet. One should note that RTM is a closed-cavity molding technology, resulting in excellent dimensional stability and surface quality [37,38].

Herein, we aimed to optimize the bonding strength of the BFs/EP interface by modifying the BFs using NaOH and two types of silane coupling agents, i.e., KH550 and KH560, before pre-impregnation. Moreover, BFs-reinforced EP composites were fabricated by using RTM, and morphology, interfacial bonding, and mechanical and thermal behavior were studied in detail. The influence of surface modification on surface chemistry and morphology of BFs was systematically investigated. In addition, the impact resistance mechanism of BFs-reinforced EP composites has been proposed.

## 2. Materials and Methods

### 2.1. Materials

Bamboo fibers (BFs) were obtained from Haibosi Co. Ltd., Fujian, China. The morphology, diameter distribution and tensile strength of BFs are shown in Figure S1. Bisphenol A epoxy resin (epoxy value: 0.51) was purchased from Xingchen Synthetic Material Co., Ltd., Nantong, China. The curing agent, methyltetrahydrophthalic anhydride (MTHPA, JHB-591A), was purchased from Jinshi Photoelectric Material Co., Ltd., Dalian, China. The promoter, 2-methylimidazole, was purchased from Chuzhou Huisheng Electronic Materials Co., Ltd., Anhui, China. The coupling agents, KH550 (boiling point: 217 °C) and KH560 (boiling point: 290 °C), were obtained from Yaohua Chemical Co., Ltd., Shanghai, China. Sodium hydroxide (NaOH, AR), acetic acid (AR), and ethanol (AR) were purchased from Tianda Chemical Reagent Co., Ltd., Tianjin, China.

### 2.2. Surface Modification

The BFs were separately treated with NaOH solution and silane coupling agent (KH550 and KH560) solution. For NaOH treatment, the dried BFs were immersed in 0–5 wt.% NaOH for 12 h at 25 °C, followed by washing with deionized water. For coupling agent treatment, the pH of the ethanol was first adjusted to 4.5–5.5 by adding acetic acid. Then, 2 wt.% coupling agent and BFs were added in the above solution at 25 °C for 2 h. The liquid-to-solid weight ratio was 20. The surface-modified BFs were dried at 103 °C for 12 h and named as raw-BFs, *x*-NaOH-BFs, KH550-BF, and KH560-BF, where x represents the concentration of NaOH solution.

### 2.3. Fabrication of BFs-Reinforced EP Composites

**Figure 1:**
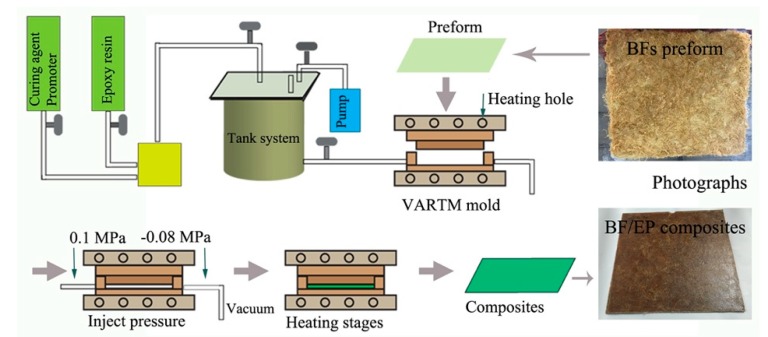
Schematic illustration of the bamboo fibers (BFs)-reinforced epoxy resin (EP) composite fabrication by resin transfer molding (RTM).

Briefly, 250 g of BFs were evenly placed in a cold pressure of 10 MPa for 12 h to obtain a 5 mm thick BFs preform at a random orientation state. Then, the preform was placed in a VARTM mold (Beijing Honghu Lion Technology Development Co., Ltd., Beijing, China) and the resin, including 1000 g of bisphenol A epoxy resin, 735 g of curing agent, and 10 g of promoter, were added to the tank and mixed evenly under mechanical agitation before closing the mold. The vacuum of the mold and tank was adjusted to −0.08 MPa for 0.5 h and the injection pressure was set at 0.1 MPa. After the resin was completely injected, the mold was stepwise heated and the temperature was sequentially increased to 60 °C, 80 °C, 100 °C, and 120 °C and maintained for 1 h at each step. Then, the mold was cooled down to obtain BFs-reinforced EP composites with the BF content of 30–35 wt.% and with the density of 1.19–1.25 g/cm³. The schematic illustration of the composite fabrication by RTM is presented in Figure 1.

### 2.4. Characterization

#### 2.4.1. Chemical Functional Groups

The surface functional groups were analyzed by using Fourier transform infrared spectroscopy (FTIR, NICOLET 6700, Thermo Fisher Co., Ltd., Agawam, MA, USA). FTIR patterns were recorded in the range of 4000–400 cm^−1^ and the resolution was 4 cm^−1^.

#### 2.4.2. Morphological Analysis

Scanning electron microscopy (SEM, JSM-7500F, Japan Electronics Corporation, Tokyo, Japan) was employed to observe the morphology of BFs-reinforced ER composites. Moreover, the atomic force microscopy (AFM, Dimension Icon, Bruker Co., Ltd., Karlsruhe, Germany) was utilized to investigate the surface microstructure of BF by using the tapping mode. The scanning rate was 0.999 Hz and the scanned area was 5 μm × 5 μm.

#### 2.4.3. Mechanical Characterization

Before the test, the tensile samples were processed by using dumbbell type sample machine (XXZ-12, Chengde Jinjian Testing Instrument Co., Ltd. Chengde, China), and the bending and impact samples were processed by using profile sample machine (XXZ-II, Jinjian Testing Instrument Co., Ltd. Chengde, China). Tensile tests were carried out in accordance with ASTM D 638 standard. The tensile specimens (165 mm × 19 mm) possess a standard dumbbell shape with a median width of 13 mm. The tensile specimens were strained at a tensile rate of 5 mm/min. The flexural strength was analyzed according to ASTM D 790 standard, where the support span was 160 mm and the crosshead speed was 1 mm/min. The dimensions of the flexural sample were 176 mm × 14 mm × 5 mm. Both tensile and flexural tests were performed on a universal testing machine (REGER Instrument Co., Ltd., Chengde, China). Herein, five independently measured values were averaged out to report the tensile and flexural strength values. The impact strength was measured according to ASTM D-256 standard by using a ZBC1400-B pendulum impacting tester (Chengde Mechanical Instrument Co., Ltd., Chengde, China). Herein, ten independently carried out impact tests were utilized to obtain a statistically reliable dataset. The dimensions of the impact specimen were 63.5 mm × 12.7 mm × 5 mm, the pendulum energy was 5.5 J, and the pendulum rate was 3.5 m/s.

#### 2.4.4. Dynamic Mechanical and Creep Analysis

Dynamic mechanical and creep behavior were analyzed by using a dynamic mechanical analyzer (DMA242, Netzsch Instrument Co., Ltd., Bavaria, Germany) under a single cantilever mode. The dimensions of the sample were 35 mm × 12 mm × 4 mm. Each set of tests was repeated thrice. The dynamic mechanical properties were assessed with a single cantilever mode under an amplitude of 50 μm and a frequency of 1 Hz in the temperature range of 25–150 °C. The samples were heated at a heating rate of 3 °C/min and the creep behavior was studied at 30 °C and 60 °C. The creep and recovery behaviors were investigated by maintaining an external load of 2 MPa for 0.5 h, followed by a gradual release in 0.5 h.

#### 2.4.5. Differential Scanning Calorimetry Analysis

Differential scanning calorimetry (DSC) curves were recorded by using Netzsch instrument (DSC-204, Bavaria, Germany). The sample (5–10 mg) was heated to 40–260 °C at a heating rate of 5 °C/min under constant nitrogen flow (50 mL min^−1^).

## 3. Results and Discussion

### 3.1. Chemical Characterization of BFs Surface

**Figure 2:**
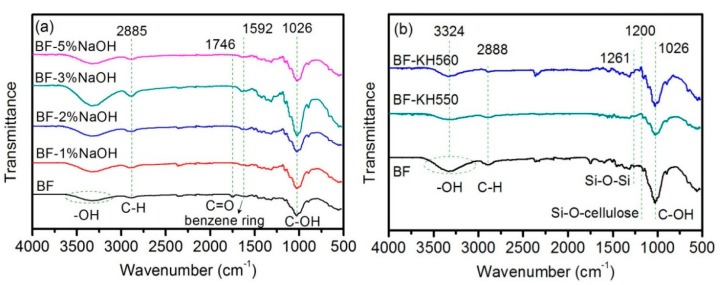
FTIR spectra of (**a**) NaOH-treated BFs and (**b**) coupling agents treated BFs.

FTIR spectra of as-received BFs and surface-modified BFs, using NaOH and coupling agents, are shown in Figure 2. The FTIR spectrum of as-received BFs exhibited a broad peak at 3665–3019 cm^−1^, which can be assigned to –OH of cellulose and lignin, which represents the stretching vibrations. Moreover, the peak at 1026 cm^−1^ corresponds to C–OH of cellulose. Compared to the as-received BFs, the –OH peak of NaOH-treated BFs is obviously stronger, which indicates more –OH exposed on the surface of the BFs due to the physical adhesion between fiber bundles was deteriorated.

Moreover, the degree of hydrolysis and peeling reaction increased with increasing NaOH concentration (Figure S2), reducing the degree of cellulose polymerization. Therefore, absorption peaks of –OH and C–OH gradually became stronger, followed by a significant decline. Moreover, the peak at 2997–2806 cm^−1^ corresponds to C–H, which represents the bending vibrations. The C=O peak of lignin, hemicellulose, and cellulose typically occurs at 1746 cm^−1^ (Figure 2a) [32]. The benzene ring skeleton of lignin (weak peak) was observed at 1592 cm^−1^, which gradually weakened and eventually disappeared with the increase of NaOH concentration. These results reveal that NaOH can remove some components from the fiber, such as hemicelluloses and lignin-like impurities [39]. However, the change in peaks is found to be irregular because the dissolved lignin and hemicellulose are re-adsorbed on BFs surface due to the hydrogen bonding of cellulose. In general, an optimal NaOH concentration is required to effectively remove the lignin and hemicellulose components and attain pure BFs.

**Figure 3:**
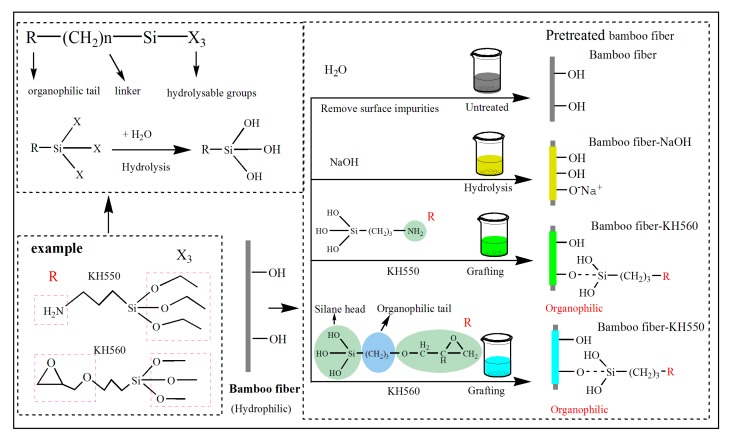
Schematic illustration and reaction mechanisms of different surface modifications.

In the case of silane-based coupling agents, the –OH peak intensity (3324 cm^−1^) was significantly decreased due to the condensation reaction between –Si(OH)_3_ of hydrolyzed coupling agents and –OH of BFs surface, resulting in Si–O–Si (1261 cm^−1^) and Si–O–cellulose (1200 cm^−1^) peaks (Figure 2b) [32]. In addition, the coupling agent, grafted on the surface of BFs, exhibited a self-polycondensation reaction. When the self-polycondensation reaction had a higher degree, the number of disappearing –OH groups was more than the surface-introduced –OH groups (reaction mechanism is shown in Figure 3). Moreover, the change in CH_2_ absorption peak (2888 cm^−1^) is mainly caused by the alkyl chains of coupling agent. The weakening of C–OH peak, corresponding to cellulose, also confirms the presence of coupling agent on the BFs surface. In addition, a weak absorption peak at 1200 cm^−1^ can be ascribed to Si–O–cellulose bond, indicating chemical bonding between silanol groups and BFs [40].

### 3.2. Morphological Analysis

**Figure 4:**
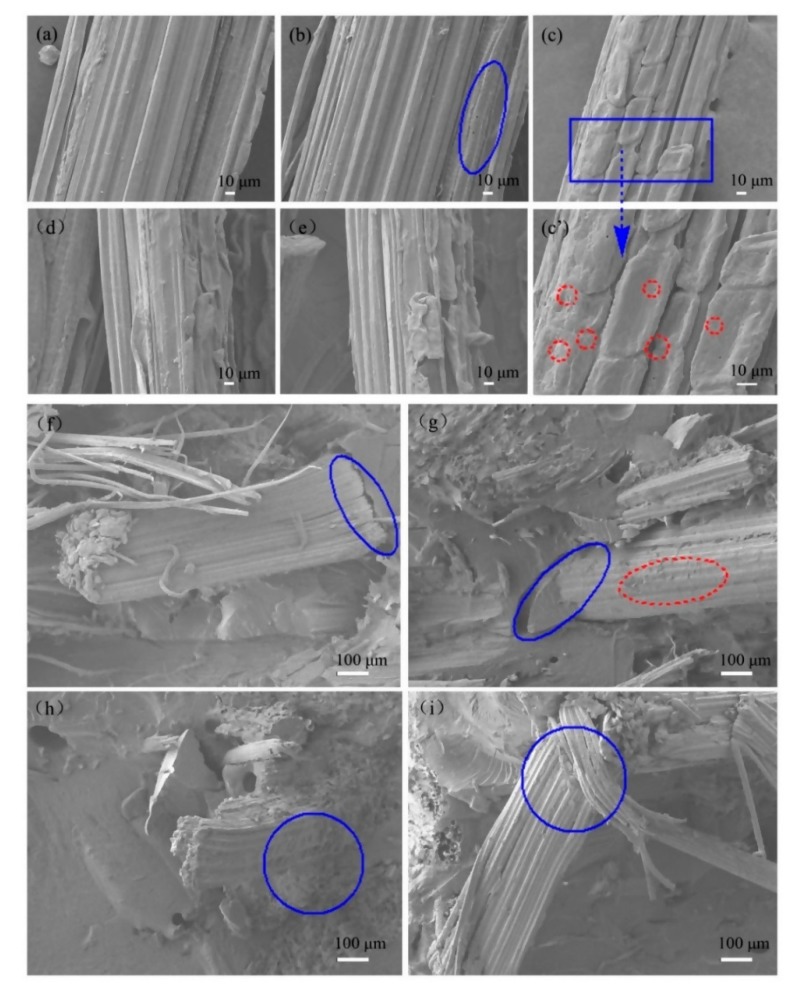
SEM images of surface-modified BFs and BFs/EP composites: (**a**) as-received BFs, (**b**) 2% NaOH-BFs, (**c**) 5% NaOH-BFs, (**d**) KH550 BFs, (**e**) KH560 BFs, (**f**) raw-BFs/EP, (**g**) 2% NaOH-BFs/EP, (**h**) KH550-BFs/EP, and (**i**) KH560-BFs/EP.

Figure 4a–e shows SEM images of as-received and surface-modified BFs. The as-received BFs exhibited a clear and regular textured surface (Figure 4a), whereas some pores were observed on BFs surface after NaOH treatment (2 wt.%). Moreover, the pore structure of the BFs surface was more clearly visible with increasing NaOH concentration to 2 wt.%. In addition, some grooves with a large undulation were observed on BFs surface in the axial direction (Figure 4a and Supplementary Materials, Figure S1), which can be ascribed to the hydrolysis and peeling reaction of BFs in NaOH solution (as discussed in Section 3.1 and Figure S2). When the concentration of NaOH increased to 5 wt.%, the roughness of the BFs surface was further increased, and showed severely fibrillated (Figure 4c’). This is mainly ascribed to the fibers that are partially peeled off from the surface due to the dissolution of the primary wall [32].

In the case of silane-treated BFs (Figure 4d–e), the clear and regular texture of BFs surface partially disappeared and some particulates adhered to the surface of BFs, exhibiting a distinct layered hierarchical structure. However, obvious differences exist in the morphology of KH550-treated and KH560-treated BFs. For instance, compared with KH560-treated BFs, the layered agglomerates on the surface of KH550-treated BFs were significantly reduced because BFs surface is rich in hydroxyl groups and these groups form a hydrogen bond, interacting with silanol groups. At the same time, the detached impurities were re-adsorbed on BFs surface under the action of coupling agent. Since two coupling agents have different molecular chains and chemical bonds, there are differences in the structure of surface agglomerates.

Figure 4f–i present the fracture morphology of BFs/EP composites, where BFs were modified by NaOH and treated with coupling agents. In the case of raw-BFs/EP composites, the significant interfacial peeling indicates poor adhesion between as-received BFs and EP resin. However, the interfacial peeling was significantly reduced after NaOH treatment, which can be ascribed to the lower content of impurities and reduction of detached components on BFs surface. Moreover, the hydrogen bonding the between BFs surface and EP matrix is enhanced due to the exposure of hydroxyl groups on the BFs surface during hydrolysis. In the case of KH550-treated BFs, we have not observed any obvious interfacial peeling between BFs and EP matrix, however, some agglomerates were observed on BFs surface (Figure 4h). One should note that the chemical interaction at the interface of two-phase material is stronger than the hydrogen bond interaction after KH550 treatment. In addition, the surface impurities exert an excellent bonding effect under the action of the coupling agent. In the case of KH560-treated BFs, we can observe visible interfacial peeling, however, no obvious agglomerates were observed on BFs surface. (Figure 4i). This can be attributed to the epoxy end group of KH560, which undergoes a ring-opening reaction under acidic conditions during the BFs modification process and increases the length of the KH560 molecular chain. During the RTM process, the molecular chain of the coupling agent migrates under the action of applied pressure and adversely affects the interfacial adhesion between BFs and EP.

**Figure 5:**
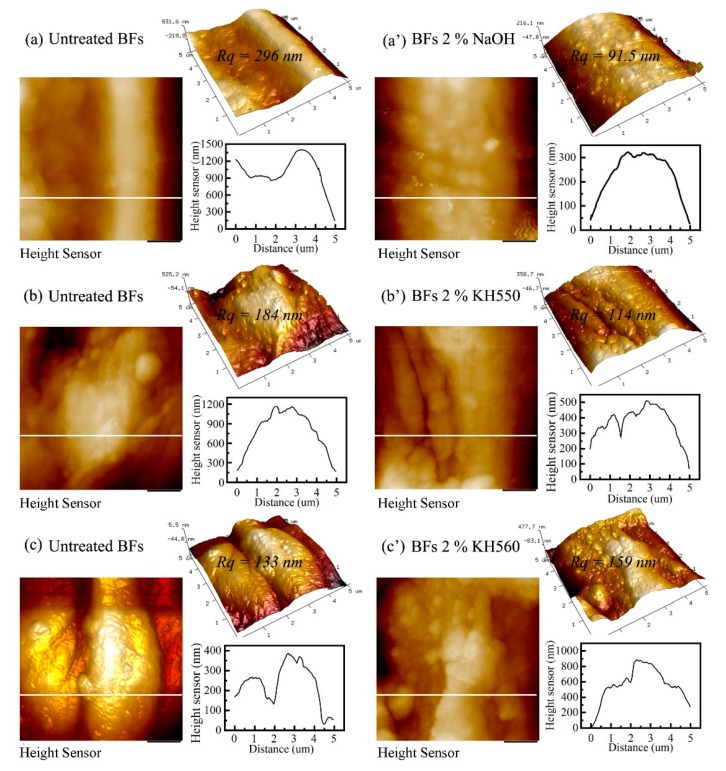
AFM images, height curves, and surface roughness of as-received and surface-modified BFs: (**a**) as-received BFs, (**a**′) 2% NaOH-BFs, (**b**) as-received BFs, (**b**′) 2% KH550-BFs, (**c**) as-received BFs, and (**c**′) 2% KH560-BFs.

Moreover, the surface roughness of BFs plays a crucial role in interface bonding. To understand the influence of surface roughness on interfacial properties, the morphological changes before and after surface modification were observed by AFM. Figure 5 shows that the roughness of BFs is decreased after NaOH and KH550 modification, however, KH560-treated BFs exhibited a higher roughness. One should note that the surface agglomerates were detached during the hydrolysis process, resulting in a lower roughness. Compared with the untreated BFs (Figure 5a), NaOH-treated BFs (Figure 5a’) exhibited significant fluctuations in the height curve due to the presence of surface pore, as shown in Figure 4b. Compared with KH550-treated BFs, the surface of KH560-treated BFs contains more agglomerates (Figure 4d,e), leading to more fluctuations in the height curve (Figure 4). Overall, AFM results indicate that although the surface roughness of BFs benefits the interfacial bonding strength, it is still weaker than the chemical bond interaction. Therefore, the interfacial properties are mainly influenced by chemical bonding interactions.

### 3.3. Mechanical Characterization

**Figure 6:**
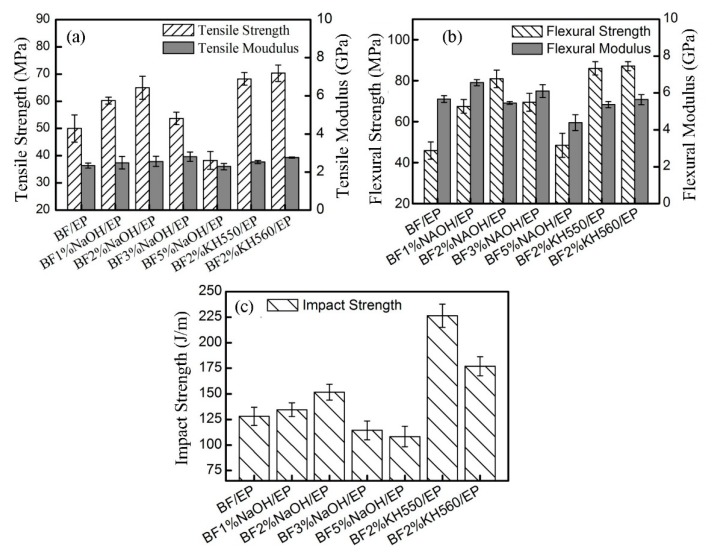
The mechanical properties of BFs/EP composites: (**a**) tensile strength and modulus, (**b**) flexural strength and modulus, and (**c**) impact strength.

Figure 6 shows the mechanical properties of BFs/EP composites. In the case of NaOH-modified BFs/EP composites, the tensile, bending, and impact strengths initially increased with increasing NaOH concentration, followed by a gradual decrease. The 2 wt.% NaOH-modified BFs/EP composite rendered optimal tensile, flexural, and impact strength of 65 ± 4.25 MPa, 80.87 ± 4.32 MPa, and 151.74 ± 7.78 J/m, respectively, corresponding to an increase of 30%, 76.19%, and 18.5% than raw-BFs/EP composites. It is worth noting that NaOH treatment effectively modified the microstructure of BFs due to hydrolysis and peeling reactions. The increased surface roughness facilitated the mechanical interlocking between BFs and EP matrix [15,41]. The mechanical strength of the 2 wt.% NaOH-modified BFs/EP composite is better than that of the untreated, which shows that the hydrogen bonding interaction is the main factor to improve its interface properties. However, the higher concentration of NaOH destroyed the structure of BFs and led to inferior toughness and poor tensile strength (Figure S1). The peeling reaction (Figure S2) is also responsible for the degradation of the mechanical strength of BFs, contributing to the deterioration of the mechanical properties of the BFs/EP composites. In addition, the dense air holes and groove structure on BFs surface act as interfacial defects due to the applied pressure during RTM. These air holes may turn into large cracks and accelerate the rupture of BFs/EP composites. Moreover, the air holes limit the interfacial interaction between BFs and EP, resulting in ineffective resin penetration. Therefore, compared with the as-received BFs, the mechanical performance of 5 wt.% NaOH-modified BFs/EP composites was significantly reduced.

In the case of silane-treated BFs/EP composites, the mechanical properties were significantly improved compared to raw-BFs and NaOH-modified BFs/EP composites. The tensile properties (strength: 70.33 ± 3 MPa; modulus: 2.76 ± 0.03 GPa) and flexural properties (strength: 87.06 ± 2.25 MPa; modulus: 5.64 ± 0.28 GPa) of KH560-modified BFs/EP composites are better than KH550-modified BFs/EP composite (tensile strength: 68.25 ± 2.32 MPa; tensile modulus: 2.53 ± 0.08 GPa and flexural strength: 85.98 ± 3.24 MPa; flexural modulus: 5.37 ± 0.16 GPa), however, the impact strength (177.17 ± 9.28 J/m) of KH560-modified BFs/EP composite is significantly lower than KH550-modified BFs/EP composite (226.37 ± 11.37 J/m). Compared with raw-BFs/EP composites, the tensile, flexural and impact strength of KH550-modified BFs/EP composites have exhibited an increase of 36%, 86.9%, and 76.6%, respectively. Compared with 2% NaOH-BFs/EP composites, the tensile, flexural and impact strengths were increased by 4.6%, 8.6%, and 48.7%, respectively.

One should note that KH550 treatment resulted in strong chemical bonding between BFs and EP matrix (mechanism as shown in Figure 3). In addition, the layered agglomerates on BFs surface improve the surrounding contact region and enhance the molecular interaction between BFs and EP matrix [41]. Consequently, compared with the hydrogen bonding, the strong chemical bonding of two-phase polymer effectively enhances the mechanical properties of the composite.

**Figure 7:**
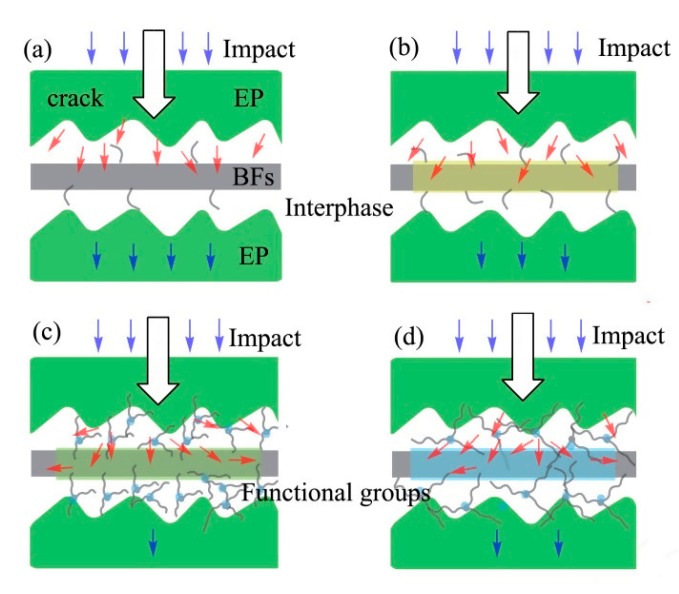
Schematic illustration of the impact resistance of (**a**) raw-BFs/EP, (**b**) NaOH-BFs/EP, (**c**) KH550-BFs/EP, and (**d**) KH560-BFs/EP composites.

Figure 7 shows the impact resistance mechanism of BFs/EP composites. The modified BFs can effectively transfer the stress concentration during the impact test [42]. Figure 7a shows that the raw-BFs/EP composites exhibit poor interfacial adhesion and the crack tip can directly contact the BFs surface, resulting in lower impact resistance. In the case of 2% NaOH-BFs/EP composites, the two-phase polymer possesses excellent mechanical interlocking. As a result, part of the stress is dispersed and consumed due to the strong hydrogen bonding during the impact process.

Furthermore, silane-modified BFs rendered lower surface polarity than as-received BFs and NaOH-modified BFs, which promoted the interfacial adhesion between BFs and EP matrix. In the case of KH550-modified BFs (Figure 7c), the interlocking between BFs and matrix is strong due to the presence of thin layer surface agglomerates, which can effectively transfer the stress concentration and alter the force direction from the crack to the EP phase [43]. In addition, a strong chemical bond exists between KH550-modified BFs and EP due to network interdiffusion, which can effectively disperse the stress and consume excessive energy, resulting in increased impact strength. Moreover, oxygen-based surface functional groups on KH560 can form strong chemically connection with EP during the curing process [41]. Compared with KH550-modified BFs, the grafting ratio between KH560 and BFs is low and the interfacial adhesion between KH560-modified BFs and EP matrix is relatively weak (Figure 4). Therefore, the impact strength of KH560-modified BFs/EP composites is degraded.

### 3.4. Dynamic Mechanical Analysis

**Figure 8:**
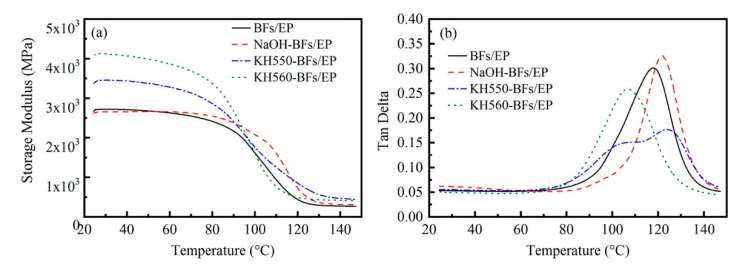
Dynamic mechanical properties of BFs/EP composites: (**a**) storage modulus (E’) and (**b**) loss factor (tan δ).

Figure 8 shows the storage modulus (E’) and tan δ versus temperature plots of BFs/EP composites. In the case of raw-BFs/EP composites, E’ decreased with increasing temperature due to the movement of the EP molecular motion unit. In a low-temperature range (25–40 °C), the rigidity of BFs/EP composites depends on the density and strength of BFs [44]. The rigidity of BFs/EP composites is also related to the interface characteristics between the mixtures. The introduction of BFs has a limitation on the movement of molecular chains of EP in a high-temperature range of 80–120 °C. Therefore, the rigidity of BFs/EP composites is mainly determined by BFs. The BFs/EP, NaOH-BFs/EP, KH550-BFs/EP, and KH550-BFs/EP composites exhibited an E’ value of 2664, 2618, 3377, and 4091 MPa, respectively, at room temperature, exhibiting a significantly large difference, which can be related to the molecular structure, aggregation state, and molecular motion unit of the polymer. After chemical modification of BFs, E’ of modified BFs/EP composites was significantly increased, which can be ascribed to the superior toughness of BFs [16].

In the case of NaOH-modified BFs/EP composites, part of the hemicellulose and lignin were removed during hydrolysis; as a result, the relative content of cellulose in BFs increased while cellulose content could provide good mechanical strength [16,45]. In addition, surface-treated BFs could reduce the free volume and decrease polymer chain mobility due to the good interfacial interaction between the BFs and matrix. Therefore, the peak intensity of *Tg* increased. Moreover, hydrogen bonding improved the interfacial properties of BFs/EP composites. In the case of the silane-modified BFs/EP composites, the *Tg* values of KH550-modified and KH560-modified BFs/EP composites are significantly different (Figure 8b), which could be ascribed to the difference in chemical bonding of coupling agents. It is also related to the interfacial compatibility between BFs and EP. In addition, at high temperature, the layered agglomerates (Figure 4) adhesion on the surface of the BFs increased the instability of the thermodynamic properties of the BFs/EP composites. Therefore, the *Tg* values of the silane-modified BFs/EP composites changed differently. In general, strong chemical bonding played a crucial role in improving the interfacial properties of BFs/EP composite [29]. One should note that the strong chemical connection originated from different modification methods and affected the overall performance of BFs/EP composites due to the transfer and consumption of additional loads.

### 3.5. Creep Analysis

**Figure 9:**
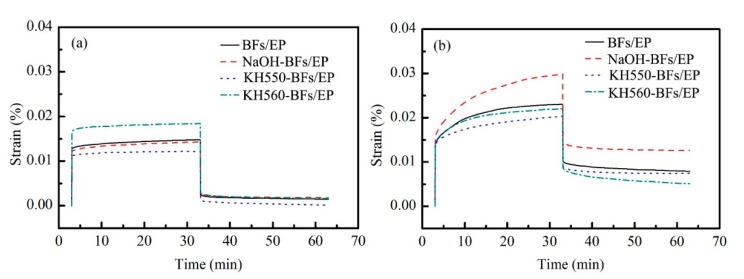
Creep-recovery curves of BFs/EP composites at (**a**) 30 °C and (**b**) 60 °C.

Figure 9 shows the creep behavior of BFs/EP composites. In Figure 9, the creep curve can be divided into three stages: elastic deformation, viscoelastic deformation, and viscous deformation. One should note that EP is a thermosetting polymer with a dense molecular structure, which implies that the EP segments are not easily moved under a constant load at room temperature. However, the movement of molecular chains is exacerbated at higher temperatures, which accelerates the creep rate of the material [44].

The total strain value of KH560-BFs/EP composite attained a maximum value at 30 °C, which was 0.0184%, followed by BFs/EP (0.0148%), BFs-NaOH/EP (0.0143%), and BFs-KH550/EP (0.0122%). After releasing the load for 30 min, the residual strain value of BFsKH550/EP composite exhibited a minimum value (0.0002%), corresponding to a maximum recovery rate of 98.9%, which can be ascribed to the improved toughness of EP matrix due to the addition of BFs. In addition, the dispersion state of BFs in EP matrix, interfacial bonding strength, and interaction between BFs and EP also influenced the creep properties.

The creep-recovery curves of KH550-BFs/EP composite exhibited similar behavior at 60 °C and 30 °C. However, the range of viscoelastic deformation stage is widened and the slope of creep curve is also significantly increased with the increase of temperature, resulting in a total strain value of 0.2031%, which is ~1.67 times higher than the stain value at 30 °C. These results confirm that the creep properties are significantly affected by the temperature. The creep behavior of NaOH-BFs/EP composite is more influenced by the temperature due to the presence of microporous structure on BFs surface and hydrogen bonding at the interface between two phases. One should note that the hydrogen bond weakens at higher temperatures. Furthermore, the strong chemical bonding at the interface renders a limiting effect on the movement of EP molecular chains. In addition, this chemical bond is also stable at 60 °C, contributing to the excellent interfacial compatibility between BFs and EP matrix.

### 3.6. Differential Scanning Calorimetry

**Figure 10:**
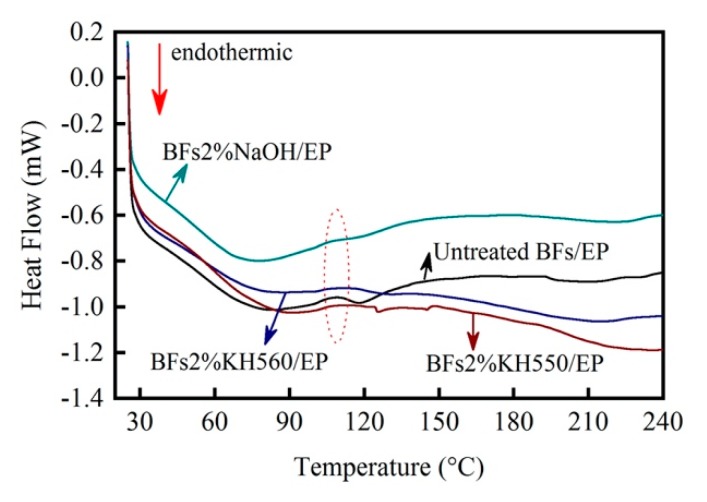
DSC curves of the BFs/EP composites in the temperature range of 30–240 °C.

Figure 10 shows the DSC curves of BFs/EP composites. In the case of raw-BFs/EP composite, a glass transition platform appeared at about 100–120 °C. Moreover, the enthalpy of BFs/EP composites linearly increased with increasing temperature. In addition, the DSC curve exhibited a large with an obvious thermal effect, which can be ascribed to the presence of excessive epoxy groups in the system. One should note that the molecular weight of the generated three-dimensional network structure was not large and the linear polymer still existed. As the reactive surface groups participated in the curing reaction of EP by chemical action, adsorption, and steric hindrance, the heat transfer behavior of composite was significantly influenced, resulting in a distinct endothermic peak at ~108 °C. When the temperature exceeded 120 °C (the highest temperature during RTM hot pressing), the change in the enthalpy curve exhibited small fluctuations, indicating that BFs/EP composite has a reduced sensitivity to temperature.

In the case of the NaOH-modified BFs/EP composite, the glass transition platform became narrow and the exothermic peak (~108 °C) was obviously weakened, indicating the influence of morphological structure on curing mechanism of EP. In addition, the BFs/EP composite had a stress history due to the injection pressure during RTM processing. The stress history was released in the form of exothermic thermal expansion in the glass transition zone, corresponding to the appearance of exothermic peaks. The DSC curve of the NaOH-modified BFs/EP composite exhibited a significantly reduced slope in the glass transition zone, which indicates that the NaOH-modified BFs effectively transferred the stress.

In the case of the silane-modified BFs/EP composites, the enthalpy exhibited a downwards trend after 108 °C, indicating the influence of chemical bonding between BFs and EP on *Tg*. In addition, the molecular cross-linking network of the silane-modified BFs/EP composites was rearranged after heating. Therefore, the silane-modified BFs/EP composites are not easily expanded during the heating process. However, the coupling agent decomposed at a higher temperature and the enthalpy curve decreased. The enthalpy curve of the KH560-modified BFs/EP composite exhibited an obvious downward trend, which can be attributed to the ring-opening reaction of remaining epoxy end groups during hydrolysis. In general, the surface physicochemical characteristics of BFs play a critical factor and influence the *Tg* and enthalpy of BFs/EP composites.

## 4. Conclusions

In summary, BFs/EP composites were successfully prepared by surface modification and the RTM process. FTIR and SEM results revealed that 2% NaOH could effectively improve the interface properties due to the synergistic effect of hydrolysis and peeling reaction of BFs. Moreover, the coupling agents were also grafted to the surface of BFs. In the case of KH550-BFs/EP and NaOH-BFs/EP composites, we have not observed any obvious interfacial peeling, whereas KH560-BFs/EP composites exhibited unobvious interfacial peeling. AFM results indicate that the surface roughness of BFs is not a determining factor for interfacial bonding.

Furthermore, compared with the raw-BFs/EP composites, mechanical properties of NaOH- and the silane-modified BFs/EP composites were significantly improved. KH550-BFs/EP composite exhibited the optimal tensile, flexural and impact strengths of 68 MPa, 86 MPa, and 226 J/m, respectively, which are 36%, 86.9%, and 76.6% higher than the corresponding strength value of the raw-BFs/EP composites. Compared with the hydrogen bonding, the chemical bonding of two-phase polymer effectively enhanced the mechanical properties by efficiently transferring the concentrated stress, which explains the mechanism of enhanced impact resistance. Moreover, dynamic mechanical analysis and creep analysis confirmed that strong chemical bonding played a crucial role in improving the overall performance of the BFs/EP composites. DSC results indicated that the glass transition platform of the BFs/EP composites appeared at about 100–120 °C. The surface-treated BFs could improve the thermal transition behavior of composites, which shows the good interfacial interaction between the BF and polymer–matrix could decrease polymer chain mobility.

Therefore, the surface-modified BFs/EP composites showed excellent interfacial bonding, mechanical properties, and thermal stabilities, which provide potential applications in the field of structural materials.

## Supplementary Materials

The following are available online at https://www.mdpi.com/2073-4360/11/12/2107/s1: Figure S1, (a) the digital and (b) optical images of bamboo fibers (BFs) (c,d) size distribution and (e) the tensile strength of bamboo fibers modified with different treatments; Figure S2: the alkaline degradation mechanism of cellulose, where ether bond is located at the β position of the negatively charged group.
