# Supplementary material for: Surface Modification of Bamboo Fibers to Enhance the Interfacial Adhesion of Epoxy Resin-Based Composites Prepared by Resin Transfer Molding
Source: Polymers (Basel). 2019 Dec 15;11(12):2107. doi: 10.3390/polym11122107 (PMC6960662; doi:10.3390/polym11122107)
Supplement: Supplementary file 1 [file polymers-11-02107-s001.pdf]

polymers

MDPI

Supporting information

Surface Modification of Bamboo Fibers to Enhance the Interfacial Adhesion of Epoxy Resin-Based Composites Prepared by Resin Transfer Molding

Dong Wang [1,†], Tian Bai [1,†], Wanli Cheng [1], Can Xu [1], Ge Wang [2], Haitao Cheng [2] and Guangping Han [1,*]

[1] Key Laboratory of Bio-based Material Science and Technology (Ministry of Education), Northeast Forestry University, Harbin 150040, China; sdzcwangdong@nefu.edu.cn (D.W.); baitian@nefu.edu.cn (T.B.); nefucwl@nefu.edu.cn (W.C.); xucan@nefu.edu.cn (C.X.)
[2] International Centre for Bamboo and Rattan, Beijing 100102, China; wangge@icbr.ac.cn (G.W.); htcheng@icbr.ac.cn (H.C.)
† There two authors contributed equally.
* Correspondence: guangping.han@nefu.edu.cn; Tel.: +86-451-821-91938

This Supporting information document contains two figures and two characterization methods (S1-S3).

Figure S1 shows the morphology, size distribution and tensile strength of bamboo fibers (BFs). The clear surface texture of BFs can be readily observed (Figure S1b). The diameter of BFs ranged from 50.4 to 687.2 μm with an average value of 247.4 μm. The length of BFs ranged from 2.2 to 26.5 cm with an average value of 10.4 cm.

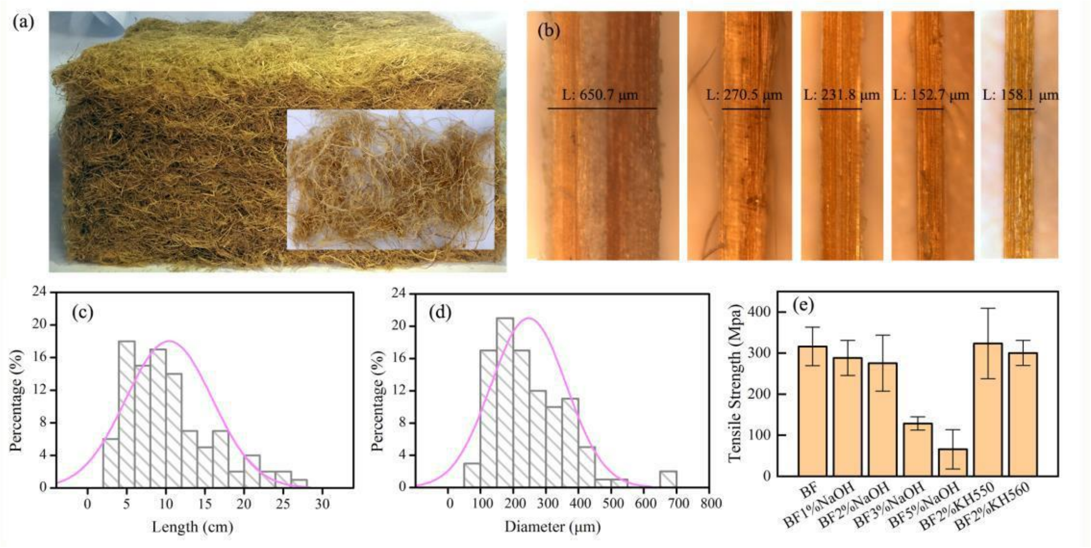

Figure S1. This (a) The digital and (b) optical images of bamboo fibers (BFs) and (c-d) size. distribution (e) the tensile strength of BFs modified with different treatments.

The alkaline degradation reaction of cellulose is mainly divided into two steps, the peeling reaction and the termination reaction, as shown in Figure S1 [2].

Peeling reaction: (1)-(2) aldosterone interconversion; (2)-(3) β-alkyl oxygen elimination reaction, glycoside bond is broken and one end group is removed; (3)-(4) tautomerization to form dicarbonyl derivatives; (4)-(5) carbon-oxygen π bond C(3) = O or C(2) = O is added by H_2O to form the gem-diols; (5)-(6) or (7) isomerization to form α-isomeric acid (6) or β-isomeric acid (7).

Stop reaction: (1)-(8) aldehyde to enol; (8)-(9) enol to ketone; (9)-(10) C(2)= O addition reaction with H_2O to form the gem-diols; (10)-(11) or (12) molecular rearrangement of gem-diols to form a carboxylic acid, and finally form an α-metaconic acid terminal group cellulose (11) or β-metabolic acid terminal cellulose (12) with a P-π conjugated system.

The glycosidic bond of the cellulose partially cleaves under alkaline conditions, resulting in a new reducing end group. Also, the cellulose glucose end group is converted into fructose end group, decreasing the degree of cellulose polymerization caused by the hydrolysis reaction. As the reaction proceeds, the glucosyl groups, with a reducing end group, gradually fall until a stable reaction is attained, where the cellulose end group is converted into a meta-saccharide group (peeling reaction). Moreover, hemicellulose and lignin are partially or completely dissolved in an alkaline solution and the rate of hemicellulose removal is much higher than the lignin. However, when the reaction proceeds to a certain extent, the rate of hemicellulose dissolution becomes lower than the lignin.

As the glycosidic bond of fructose end group exists in β position of C=O, the elimination reaction can occur in the alkaline solution due to the strong electron withdrawing group (Figure S2). For β-alkoxycarbonyl structure in cellulose, the alkoxy group can be rapidly eliminated under alkaline conditions, whereas HO-(G)n-1 attains a new reducing terminal group and continues the above reaction till completion be taken off one by one.

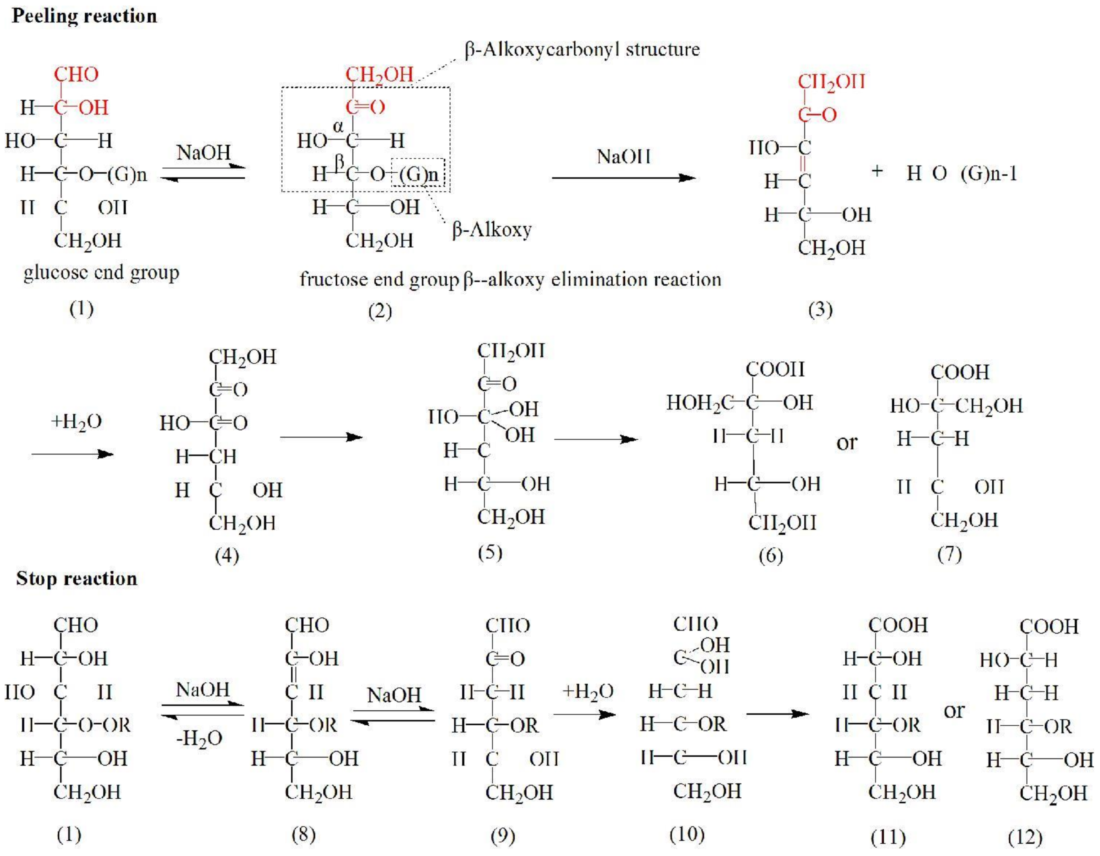

Figure S2. The alkaline degradation mechanism of cellulose, where ether bond is located at the β. position of the negatively charged group.

Characterization

Optical microscopy images of the BFs were created and the dimensions of BFs were determined by using a Nikon imaging system (Ci-E/Ci-L/Ci-S, Electron Optics Labotatory Co., Tokyo, Japan).

The mechanical property was tested according to the literature method [1]. Before the tensile test, the BFs were attached on a paper with epoxy adhesive. Next, samples were dried at 80 °C for 6 h to cure the epoxy adhesive. Ultimately, the tensile strength of single BFs was measured by a

microelectronic universal testing machine (WDW-20, Shenzhen REGER Instrument Co. Ltd., China). Noted that the BFs used in this study had an average diameter about 250 μm. The length of sample was cut to 40 mm and the tensile rate was 5 mm/min. The values were tested five times to ensure the reliability.

References

1. Huang, J. K.; Young, W. B., The mechanical, hygral, and interfacial strength of continuous bamboo fiber Reinforced epoxy composites. *Composites Part B: Engineering*, **2019**, *166*, 272-283, DOI: 10.1016/j.compositesb.2018.12.013.
2. Jicheng Pei, A.; Shuzheng Yang, B. *Lignocellulosic chemistry*, 4rd ed.; China Light Industry Press: Beijing, China, 2016; pp. 205-207.

© 2019 by the authors. Licensee MDPI, Basel, Switzerland. This article is an open access article distributed under the terms and conditions of the Creative Commons Attribution (CC BY) license (http://creativecommons.org/licenses/by/4.0/).
